# Effect of APOE and a polygenic risk score on incident dementia and cognitive decline in a healthy older population

**DOI:** 10.1111/acel.13384

**Published:** 2021-05-26

**Authors:** Moeen Riaz, Aamira Huq, Joanne Ryan, Suzanne G Orchard, Jane Tiller, Jessica Lockery, Robyn L. Woods, Rory Wolfe, Alan E. Renton, Alison M. Goate, Robert Sebra, Eric Schadt, Amy Brodtmann, Raj C. Shah, Elsdon Storey, Anne M Murray, John J. McNeil, Paul Lacaze

**Affiliations:** ^1^ Department of Epidemiology and Preventive Medicine School of Public Health and Preventive Medicine Monash University Melbourne Vic Australia; ^2^ Department of Genomic Medicine Royal Melbourne Hospital University of Melbourne Melbourne Vic Australia; ^3^ Department of Medicine Royal Melbourne Hospital University of Melbourne Melbourne Vic Australia; ^4^ Nash Family Department of Neuroscience and Ronald Loeb Center for Alzheimer’s Disease Icahn School of Medicine at Mount Sinai New York NY USA; ^5^ Departments of Neurology and Genetics and Genomic Sciences Icahn School of Medicine at Mount Sinai New York NY USA; ^6^ Department of Genetics and Genomic Sciences Icahn Institute for Data Science and Genomic Technology Icahn School of Medicine at Mount Sinai New York NY USA; ^7^ Florey Institute of Neuroscience and Mental Health Melbourne Vic Australia; ^8^ Melbourne Dementia Research Centre University of Melbourne Melbourne Vic Australia; ^9^ Department of Family Medicine and Rush Alzheimer's Disease Center Rush University Medical Center Chicago Illinois USA; ^10^ Berman Center for Outcomes and Clinical Research Hennepin Healthcare Research Institute University of Minnesota Minneapolis MN USA

**Keywords:** Alzheimer's disease, Apolipoprotein E, aspirin in reducing events in the elderly, cognition, cognitive decline, cumulative incidence of dementia, genome‐wide association study, polygenic risk score

## Abstract

Few studies have measured the effect of genetic factors on dementia and cognitive decline in healthy older individuals followed prospectively. We studied cumulative incidence of dementia and cognitive decline, stratified by *APOE* genotypes and polygenic risk score (PRS) tertiles, in 12,978 participants of the ASPirin in Reducing Events in the Elderly (ASPREE) trial. At enrolment, participants had no history of diagnosed dementia, cardiovascular disease, physical disability or cognitive impairment. Dementia (adjudicated trial endpoint) and cognitive decline, defined as a >1.5 standard deviation decline in test score for either global cognition, episodic memory, language/executive function or psychomotor speed, versus baseline scores. Cumulative incidence for all‐cause dementia and cognitive decline was calculated with mortality as a competing event, stratified by *APOE* genotypes and tertiles of a PRS based on 23 common non‐*APOE* variants. During a median 4.5 years of follow‐up, 324 participants developed dementia, 503 died. Cumulative incidence of dementia to age 85 years was 7.4% in all participants, 12.6% in *APOE* ε3/ε4 and 26.6% in ε4/ε4. *APOE* ε4 heterozygosity/homozygosity was associated with a 2.5/6.3‐fold increased dementia risk and 1.4/1.8‐fold cognitive decline risk, versus ε3/ε3 (*p *< 0.001 for both). High PRS tertile was associated with a 1.4‐fold dementia risk versus low (CI 1.04–1.76, *p *= 0.02), but was not associated with cognitive decline (CI 0.96–1.22, *p* = 0.18). Incidence of dementia among healthy older individuals is low across all genotypes; however, *APOE* ε4 and high PRS increase relative risk. *APOE* ε4 is associated with cognitive decline, but PRS is not.

## Graphical abstract

We studied cumulative incidence of dementia and cognitive decline in 12,978 healthy older individuals without a history of cardiovascular disease. Dementia incidence was low across all genotype groups, but APOE ε4 and high PRS still increased relative risk. APOE ε4 was associated with cognitive decline risk, but PRS was not.

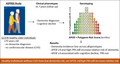

**Abbreviations**
AD: Alzheimer's disease
APOE: Apolipoprotein E
ASPREE: ASPirin in Reducing Events in the Elderly
CID: Cumulative incidence of dementia
GWAS: Genome‐wide association study
HWE: Hardy–Weinberg equilibrium
PRS: Polygenic risk score

## 1 INTRODUCTION

Few studies have measured the effect of Apolipoprotein E (*APOE*) genotypes and polygenic risk scores (PRS) on incident dementia and cognitive decline in healthy older people. The ASPREE (ASPirin in Reducing Events in the Elderly) cohort offers the opportunity to measure these effects, as recruited participants had no history of cardiovascular disease, dementia or significant physical disability at enrolment. The ASPREE study was a randomised, placebo‐controlled trial to determine whether daily low dose aspirin increased survival, free of dementia or persistent physical disability, in 19,114 healthy community‐dwelling older people (McNeil et al., 2017). In 2018, ASPREE reported that over an average 4.5 years of follow‐up, aspirin did not prolong disability‐free survival (McNeil et al., 2018a, 2018b, 2018c) or reduce the risk of dementia or cognitive decline (Ryan et al., 2020).

The *APOE* gene is the strongest genetic determinant of all‐cause dementia, especially Alzheimer's disease (AD), with the ε4 allele elevating risk and accelerating age of onset (Qian et al., 2017; Rasmussen et al., 2018; Saunders et al., 1993; Lee et al., 2018). The ε4 allele is also associated with cognitive impairment (dysfunction in episodic memory, processing speed, executive function or global cognition) in people without a dementia diagnosis (Albrecht et al., 2015; Jager et al., 2012; Reas et al., 2019; Wisdom et al., 2011). Beyond *APOE*, common disease‐associated variants identified from genome‐wide association studies (GWAS) (Harold et al., 2009; Hollingworth et al., 2011; Kunkle et al., 2019; Lambert et al., 2013; Naj et al., 2011) also modify dementia risk and can be used to calculate a polygenic risk score (PRS) (Tan et al., 2017; Chouraki et al., 2016; Cruchaga et al., 2018; Desikan et al., 2017; Escott‐Price et al., 2015; Sleegers et al., 2015; Lee et al., 2018). Individually, these common genetic variants have low effect sizes, yet when combined into a PRS can enable risk‐stratification for dementia indications beyond *APOE* genotype. There is varying evidence for whether a PRS for dementia can also predict cognitive decline (Chaudhury et al., 2019; Harris et al., 2014; Marden et al., 2016; Verhaaren et al., 2013). Incorporating both *APOE* genotypes and PRS, alongside conventional risk factors, may enable more accurate risk prediction (Licher et al., 2019; Lourida et al., 2019). This may aid development of therapeutic strategies or prevention, and advance our understanding of the genetic differences between (diagnosed) dementia and cognitive decline.

The predictive performance of PRSs for dementia requires further investigation in well‐characterised prospective studies. Predictive performance can be influenced by factors such as ethnicity, age, study recruitment criteria, clinical diagnostic criteria, neuropsychological assessments used, genotyping platform and genetic variants included (Tan et al., 2017; Chouraki et al., 2016; Cruchaga et al., 2018; Desikan et al., 2017; Escott‐Price et al., 2015; Leonenko et al., 2019; Licher et al., 2019; Lourida et al., 2019; Qian et al., 2017; Rasmussen et al., 2018; Saunders et al., 1993; Sleegers et al., 2015; Lee et al., 2018). More studies of cognitively healthy elderly individuals followed prospectively are required to assess variability and predictive accuracy. Here, we report the effects of *APOE* and PRS on incident dementia and cognitive decline among 12,978 ASPREE participants, where dementia was an exclusionary criterion at entry and adjudicated as a primary trial endpoint.

## 2 METHODS

### 2.1 Study population

Consistent with the ASPREE inclusion criteria (McNeil et al., 2018a), participants had no previous history or current diagnosis of atherothrombotic cardiovascular disease, dementia, loss of independence with basic activities of daily living or life‐threatening illness. Participants passed a global cognition screen at enrolment (>77 on the Modified Mini‐Mental State (3MS) Examination). Informed consent for genetic analysis was obtained, with ethical approval from the Alfred Hospital Human Research Ethics Committee (390/15) and site‐specific Institutional Review Boards (US).

### 2.2 Incident Dementia Diagnosis

After standardised cognition and functional measures, participants reporting memory or cognitive problems were assessed by specialists or prescribed dementia medication. Following identification of dementia triggers (3MS<78 or a drop of >10.15 points from the participant's baseline 3MS score, accounting for age and education), additional assessments were conducted, with brain imaging and laboratory analyses collected for adjudication. Each dementia trigger case was reviewed according to the ASPREE protocol for clinical adjudication (McNeil et al., 2018c; Ryan et al., 2020) by an adjudication committee consisting of geriatricians, neurologists and neuropsychologists. Dementia was diagnosed using Diagnostic and Statistical Manual of Mental Disorders, fourth edition criteria. Diagnosis date was recorded as date of trigger. Dementia cases were sub‐classified into either ‘probable AD’, ‘possible AD’ or ‘non‐AD dementia’, using the 2011 NIA‐Alzheimer's Association core clinical criteria (McKhann et al., 2011).

### 2.3 Cognitive decline

The ASPREE cognitive battery included the 3MS for general cognition, the Hopkins Verbal Learning Test‐Revised (HVLT‐R) delayed recall for episodic memory, the single letter Controlled Oral World Association Test (COWAT) for language and executive function, and the Symbol Digit Modalities Test (SDMT) to measure psychomotor speed. Accredited professionals administered assessments at baseline and year 1, followed biennially during follow‐up. As reported previously (Ryan et al., 2020) cognitive decline in participants without a dementia diagnosis was defined as a 1.5 standard deviation decline in 3MS/HVLT‐R/SDMT/COWAT compared with baseline scores, sustained over ≥2 time points.

### 2.4 Genotyping and variant analysis

Figure S1 shows a flow chart to describe how participants were included/excluded in the study. Out of 19,114 total ASPREE trial participants, 13,941 participants (73%) provided samples to the ASPREE biobank. DNA extracted from these samples was then genotyped using the Axiom 2.0 Precision Medicine Diversity Research Array following standard protocols. 12,978 samples passed quality control (12,343 Australian, 635 US) based on sex, relatedness and Non‐Finnish European ancestry. Details of the 963 samples that were excluded from the final analysis (204 QC failed and 759 based on ancestry) is provided in the supplementary material. To estimate population structure, we performed principal component analysis using the 1000 Genomes reference population (Figure S2) (Auton et al., 2015; Zheng et al., 2012). Imputation was performed using the haplotype reference consortium European panel (Das et al., 2016). Post‐imputation quality control removed variants r2 < 0.3. *APOE* genotype was measured using two directly genotyped variants (rs7412, rs429358) extracted using plink v1.9 (Chang et al., 2015)

### 2.5 Polygenic risk score

PRS was calculated using 23 common variants (15 genotyped, 8 imputed) associated with AD at genome‐wide significance that affect risk independently of *APOE* (Desikan et al., 2017; Lambert et al., 2013; Ruiz et al., 2014). PRS calculations, using plink v1.9 (Chang et al., 2015), were based on dosage (0,1,2) of SNP effect allele reported from GWAS, multiplied by effect sizes, followed by the sum of products to generate a PRS per participant (Table S1). We used the same 23 SNP PRS and the same PRS calculation methods used in recent analysis of the Rotterdam study (Lee et al., 2018). PRS distribution was divided into low/middle/high tertiles; with mean values of; low −0.56 (range −1.43 to −0.34), middle −0.20 (−0.34 to −0.06) and high 0.16 (−0.06 to 1.86) (Figure S3). Tertiles were used to ensure equal distribution of samples across PRS groups, and sufficient events numbers occurred in each group for statistical power possible (Figure S3). In addition, we sought to use the same analysis approach as the Rotterdam Study (Lee et al., 2018), where tertiles were also used.

### 2.6 Statistical analysis

To determine whether *APOE* genotype frequencies were under selective pressure due to age and/or trial inclusion/exclusion criteria, we performed Hardy–Weinberg equilibrium (HWE) testing. This compared observed genotype frequencies with those expected in a population under no selective pressure, using chi‐squared tests. We examined the cumulative incidence of dementia (CID) and cognitive decline, stratified by *APOE* genotype and PRS tertiles. We used ε3 homozygotes as a reference group for *APOE*‐stratified analysis and the low‐risk tertile for PRS‐stratified analysis. Consistent with other studies (Desikan et al., 2017; Lee et al., 2018) we combined *APOE* ε3/ε4:ε2/ε4 into a single group, and ε2/ε2:ε2/ε3 into a single group.

We estimated cumulative incidence of all‐cause dementia and cognitive decline during an average of 4.5 years of follow‐up, using the Cumulative Incidence Function (CIF) of the etm package (Allignol et al., 2011; Meister & Schaefer, 2008) in R version 3.6.0 (R Core Team, 2013). Data were censored by date of dementia diagnosis, cognitive decline, last contact or death. The age on censored date was used as a time scale in CIF model. Cumulative incidence was calculated up to 95 years, then stratified by *APOE* genotype and PRS tertiles. Dementia and cognitive decline between PRS tertiles were compared for the whole cohort and further stratified by *APOE* genotypes. The dementia and cognitive decline models were estimated independently.

We used the Fine and Gray (F&G) method of accounting for competing risk of death, and Cox proportional hazard regression model to calculate dementia hazard ratio of both models, for *APOE*, PRS and their interaction, adjusted for age at enrolment (continuous, allowing a quadratic function) and sex (Meister & Schaefer, 2008; Lee et al., 2018). We used age on censored date as a time scale in both F&G and Cox models. Hazard ratios for cognitive decline were measured the same way. To test association of *APOE* genotypes and PRS with cohort characteristics, we used a multivariable regression model with variables; age, sex, follow‐up time, education, alcohol use, smoking, diabetes mellitus, hypertension, depression (Center for Epidemiological Studies‐Depression‐10 scale), family history of dementia (father/mother/sibling), body mass index, blood pressure, cholesterol and triglycerides. Bonferroni multiple test correction at *p *= 0.002 significance was applied (0.05/17 = 0.002). We followed the Strengthening the Reporting of Observational Studies in Epidemiology (STROBE) guidelines for reporting of results, see Table S2.

## 3 RESULTS

Characteristics of the 12,978 genotyped participants are shown in Table 1. Overall, 54.8% were female, 47% had educational attainment <12 years, 3% were current smokers and 25% reported a family history of dementia at enrolment.

**TABLE 1:** Characteristics of the ASPREE cohort stratified by *APOE* genotypes and tertiles of a PRS

	All	ε3/ε3	ε3/ε4	ε2/ε2	ε2/ε3	ε2/ε4	ε4/ε4	Low PRS tertile	Middle PRS tertile	High PRS tertile
Total	12978	7800(60.0%)	2665(20.5%)	68(0.5%)	1784(14.0%)	461(3.5%)	200(1.5%)	4326(33.3%)	4330(33.4%)	4322(33.3%)
Demographics										
Age, Years	75.05(4.2)	75.12(4.3)	74.66(3.9)	74.97(3.9)	75.41(4.4)	75.28(4.4)	73.91(3.4)^a^	75.15(4.3)	75.04(4.2)	74.95(4.1)
Follow up time, Years	4.53(1.3)	4.54(1.3)	4.50(1.3)	4.63(1.3)	4.54(1.3)	4.63(1.2)	4.46(1.3)	4.51(1.3)	4.52(1.3)	4.50(1.3)
Sex										
Females	7120(55.0%)	4407(56.0)	1413(53.0%)	40(58.8%)	916(51.3%)	245(53.0%)	99(49.5%)	2385(55.0%)	2371(54.7%)	2364(54.6%)
Males	5858(45.0%)	3393(44.0)	1252(47.0%)	28(41.2%)	868(48.7%)	216(47%)	101(50.5%)	1941(45.0%)	1959(45.3%)	1958(45.4%)
Education										
<12 years	6136(47.2%)	3640(46.6%)	1249(46.9%)	27(39.7%)	886(49.6%)	230(49.9%)	104(52.0%)	2030(46.9%)	2061(47.6%)	2045(47.4%)
12–15 years	3483(26.8%)	2118(27.0%)	707(26.5%)	18(26.4%)	483(27.10%)	114(24.7%)	43(21.5%)	1137(26.3%)	1178(27.2%)	1168(27.0%)
16+ years	3359(26.0%)	2042(26.4%)	709(26.6)	23(33.9%)	415(23.3%)	117(25.4%)	53(26.5%)	1159(26.8%)	1091(25.1%)	1109(25.6%)
Alcohol Use										
Current	10353(79.8%)	6254(80.2%)	2108(79.0%)	55(81.0%)	1421(79.6%)	355(77.0%)	160(80%)	3438(79.4%)	3473(80.2%)	3442(79.7%)
Former	620(4.8%)	372(4.8%)	129(4.8%)	0(0%)	89(5.0%)	21(4.6%)	9(4.5%)	210(4.9%)	209(4.8%)	201(4.6%)
Never	2005(15.4%)	1174(15.0%)	428(16.2%)	13(19.0%)	274(15.4%)	85(18.4%)	31(15.5%)	678(15.7%)	648(15.0%)	679(15.7%)
Smoking status										
Current	394(3.0%)	244(3.1%)	83(3.1%)	2(3.0%)	44(2.5%)	11(2.4%)	10(5.0%)	115(2.6%)	137(3.2%)	142(3.2%)
Former	5339(41.2%)	3165(40.6%)	1111(41.7%)	29(42.6%)	749(42.0%)	195(42.3%)	90(45.0%)	1834(42.4%)	1738(40.1%)	1767(41.0%)
Never	7245(55.8%)	4391(56.3%)	1471(55.2%)	37(54.4%)	991(55.5%)	255(55.3%)	100(50.0%)	2377(55.0%)	2455(56.7%)	2413(55.8%)
Medical History										
Diabetes	1205(9.2%)	747(9.5%)	223(8.3%)	7(10.2%)	173(9.7%)	41(8.8%)	15(8.0%)	396(9.1%)	399(9.2%)	410(9.4%)
Hypertension	9553 (73.6%)	5773(74.0%)	1927(31.0%)	48(70.5%)	1330(74.5%)	341(7.4%)	137(68.5%)	3172(73.3%)	3202(73.9%)	3178(73.5%)
Depression	1177 (9.0%)	702(9.8%)	232(8.7%)	6(8.8%)	172(9.6%)^a^	42(9.1%)	23(11.5%)^a^	396(9.1%)	390(9.0%)	391(9.0%)
Dementia Family History+	3293(25.4%)	1824(23.4%)	846(31.7%)^b^	11(16.1%)	399(22.4%)	118(25.6%)	95(47.5%)^b^	1079(25.0%)	1053(24.3%)	1161(26.8%)^a^
Physical Examination										
Body Mass Index	27.97(4.5)	28.08(4.6)	27.62(4.4)^a^	29.60(5.4)^a^	28.09(4.4)	27.74(4.3)	27.66(5.0)^a^	27.98(4.6)	28.04(4.6)	27.90(4.5)
Systolic Blood Pressure (mm Hg)	139.46(16.3)	139.37(16.2)	139.40(16.1)	139.44(16.2)	139.95(16.4)	140.05(17.0)	138.1(15.3)	139.50(16.2)	139.62(16.4)	139.26(16.2)
Diastolic blood pressure (mm Hg)	77.17(9.9)	77.80(9.8)	77.19(10.0)	77.69(8.8)	77.40(10.2)	77.79(10.0)	76.49(10.0)	76.88(9.9)	77.51(9.9)	77.12(10.0)
Blood Biochemistry										
TC (mmol/L)	5.26(0.97)	5.28(0.96)	5.34(1.0)	4.65(0.90)^a^	5.07(0.91)^a^	5.24(0.95)	5.36(1.0)	5.27(0.98)	5.27(0.96)	5.25(0.96)
LDL (mmol/L)	3.07(0.86)	3.11(0.86)	3.17(0.89)	2.31(0.73)	2.81(0.76)	2.99(0.84)	3.22(0.91)	3.08(0.87)	3.08(0.86)	3.07(0.86)
HDL (mmol/L)	1.58(0.45)	1.58(0.45)	1.57(0.46)^a^	1.63(0.41)	1.61(0.47)^a^	1.60(0.46)	1.50(0.46)	1.59(0.45)	1.58(0.46)	1.57(0.45)
TG(mmol/L)	1.31(0.65)	1.29(0.60)	1.30(0.69)	1.54(0.62)^a^	1.38(0.71)^a^	1.39(0.76)	1.42(0.83)	1.31(0.65)	1.31(0.63)	1.32(0.66)
Disease/Trait Incidence										
Dementia	324(2.5%)	147(1.8%)	125(4.7%)	0(0%)	25(1.4%)	9(1.9%)	18(9.0%)	99(2.2%)	96(2.2%)	129(2.9%)
^d^Possible AD	176(1.35%)	88(1.1%)	60(2.2%)	0(0%)	15(0.84%)	5(1.1%)	8(4.0%)	58(1.3%)	55(1.3%)	63(1.4%)
^d^Probable AD	143(1.01%)	57(0.73%)	64(2.4%)	0(0%)	8(0.44%)	4(0.8%)	10(5.0%)	39(0.9%)	39(0.9%)	65(1.5%)
^d^Non‐AD‐related dementia	5(0.03%)	2(0.02%)	1(0.03%)	0(0%)	2(0.11%)	0(0%)	0 (0%)	2(0.04%)	2(0.04%)	1(0.02%)
Cognitive Decline^c^	1598 (12.6%)	896(11.1%)	385(14.4%)	8(1.4%)	208(11.6%)	67(6.9%)	34(17.0%)	518(12.0%)	534(12.3%)	546(12.6%)

Note

The numbers reported as either *n*(%) or mean(SD), +sign represents family history of dementia in either Father, mother or sibling.

Abbreviations: APOE, Apolipoprotein E; HDL, high‐density lipoprotein; LDL, low‐density lipoprotein; mm Hg, millimetre of mercury; mmol/L, Millimoles per litre; PRS, Polygenic risk score; TC, total cholesterol; TG, triglycerides.

^a^ Denotes *p* value < 0.05, as measured using a regression model with *APOE* ε3/ε3 and low PRS tertile as reference groups.

^b^ Denotes associations that remained significant after Bonferroni correction for multiple testing.

^c^ Denotes the number of samples with cognitive decline out of total 12,978 genotyped samples excluding 324 dementia cases and 326 participants with missing data.

^d^ Dementia subtype classification is performed using 2011 NIA–Alzheimer's Association core clinical criteria (McKhann et al., 2011).

Associations of cohort characteristics with *APOE* genotypes and PRS tertiles are also shown in Table 1. The only associations to survive multiple testing correction were family history of dementia in ε4 heterozygotes/homozygotes, with no cohort characteristics differing between PRS tertiles (Table 1).

We found that *APOE* genotype frequencies had deviated from HWE (chi‐square = 38, *p* < 0.001) (Table S3), with fewer ε3/ε4 heterozygotes (*N* = 2665 observed, *N* = 2723 expected) and fewer ε4/ε4 homozygotes (*N* = 200 observed, *N* = 239 expected) than expected under HWE.

During mean 4.5 years of follow‐up (interquartile range 2.1 to 5.7 years, 2779 person‐years), we observed 324 (2.5%) incident all‐cause dementia cases and 503 (3.8%) deaths (Tables 1 and 2). Of these, 143 were classified as ‘probable AD’ and 176 were classified as ‘possible AD’. Only 5 cases were classified as ‘non‐AD related dementia’ (Table 1). For cumulative incidence of dementia (CID), we describe results up to age 85 years, representing an approximate centre point between lower and upper age ranges of the ASPREE population at baseline (70 to 96 years). CID in ASPREE was estimated at 7.4% (CI 6.5 to 8.5).

**TABLE 2:** Cumulative incidence of dementia and death in ASPREE

Age^b^ (years)	Participants	Dementia^a^ (95% CI)	Death (95% CI)	Alive without Dementia (%)
75	6162	0.6% (0.4–0.8)	1.7% (1.3–2.1)	97.7
80	3063	2.9% (2.4–3.4)	4.8% (4.3–5.5)	92.3
85	1251	7.4% (6.5–8.5)	10.6% (9.5–11.8)	82.0
90	293	15.6% (13.6–17.8)	19.4% (17.3–21.6)	64.8
95	26	23.9% (19.1–29.7)	35.0% (29.4–41.4)	42.3

Abbreviation: CI, Confidence interval.

^b^ Longitudinal year age from baseline to the event of interest such as dementia, cognitive decline, death or alive with no dementia.

a Cumulative incidence of dementia with competing risk of death.

**FIGURE 1:**
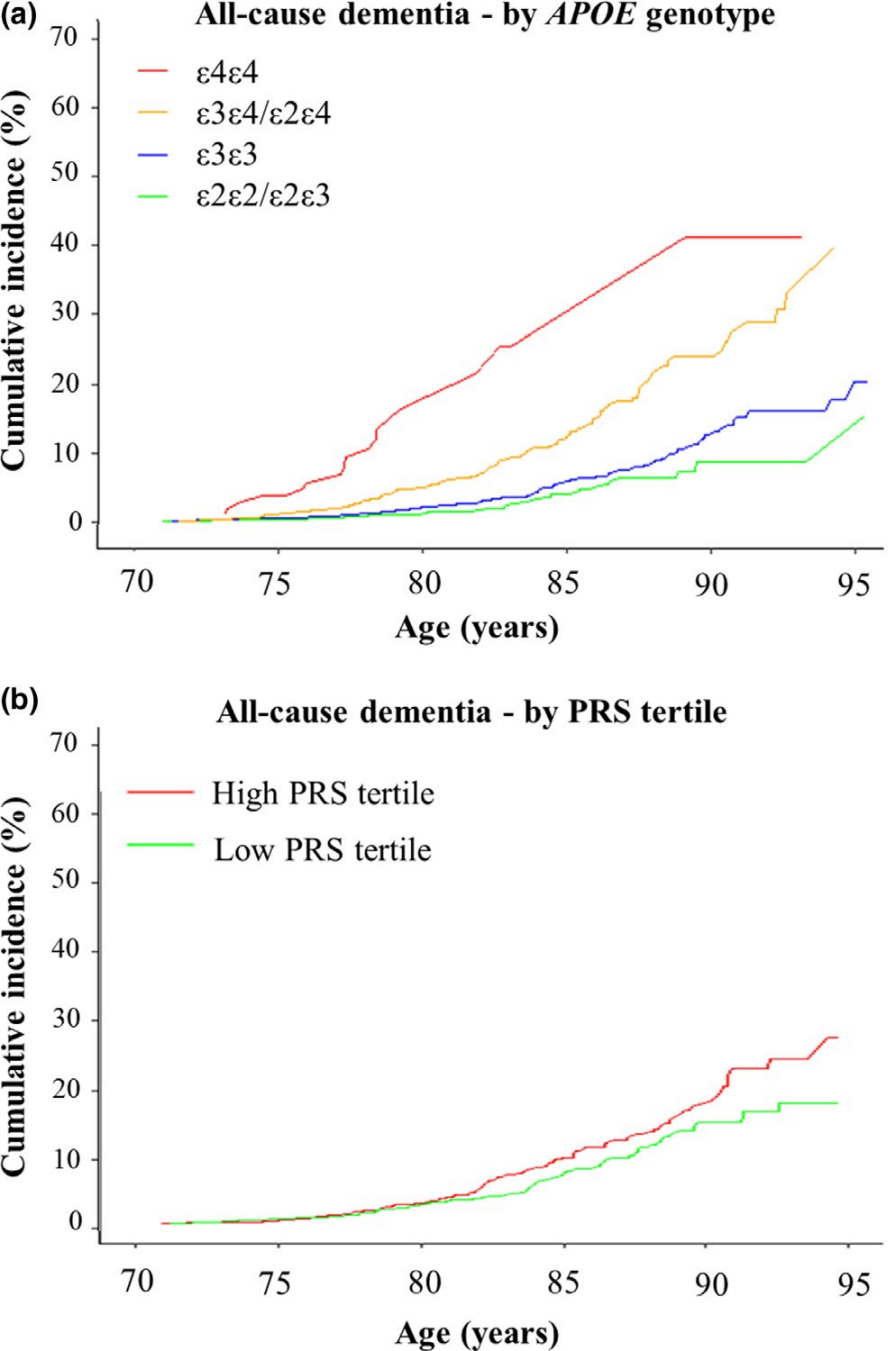
Cumulative incidencece of all‐cause dementia stratified by *APOE* genotypes and tertiles of a polygenic risk score (PRS). Cumulative incidence curves for all‐cause dementia (a) and cognitive decline (b) were calculated to age 95 years and stratified by *APOE* genotype, with mortality as a competing event. Confidence intervals and participants at risk are shown in Table S4–5

In *APOE* genotype‐stratified analysis of CID, after adjusting for covariates and death as a competing event, ε4/ε4 genotype was significantly associated with dementia risk (HR 6.38 [CI 3.8–10.7] *p* = 2.0 × 10^−12^) compared with ε3/ε3 (Table 3a). Individuals with ε3/ε4 heterozygosity were also at higher risk of dementia (HR 2.51 [CI 1.9–3.1], *p* = 1.5 × 10^−14^) compared with ε3/ε3. CID was 26.6% (CI 16.2–42.0) for ε4/ε4 homozygotes, 12.6% (CI 10.2–15.5) for ε3/ε4 heterozygotes, 5.9% (CI 4.8–7.2) for the common ε3/ε3 genotype group and 4.0% (CI 2.4–6.5) for the lower‐risk ε2/ε2:ε2/ε3 group (Figure 1a). For all *APOE* genotype‐stratified results, see Table S4.

**TABLE 3:** Cox proportional hazard ratio and risk regression models for dementia and cognitive decline risk in the ASPREE cohort

(a) Dementia	^a^No competing risk adjustment	^b^Adjusting for competing risk		
Variables	HR (95% CI)	*p* value	HR (95% CI)	*p* value
Age, years	1.16 (1.13–1.18)	0.002	1.25 (1.14–1.37)	<0.0001^c^
Sex (female)	0.76 (0.61–0.94)	0.01	0.77 (0.62 – 0.96)	0.02
*APOE* ε3ε3	Reference	‐	Reference	‐
*APOE* ε2ε2:ε2ε3	0.67 (0.43–1.02)	0.06	0.65 (0.43 −1.00)	0.05
*APOE* ε3ε4:ε2ε4	2.50 (1.97–3.16)	<0.0001^+^	2.51 (1.98 – 3.17)	<0.0001^+^
*APOE* ε4ε4	6.32 (3.86–10.34)	<0.0001^+^	6.38 (3.81–10.71)	<0.0001^+^
Low PRS tertile	Reference	‐	Reference	‐
Middle PRS tertile	1.00 (0.75–1.32)	0.98	1.00 (0.76 −1.33)	0.95
High PRS tertile	1.36 (1.04–1.76)	0.02	1.36 (1.04–1.77)	0.02

(b) Cognitive decline	^a^No competing risk adjustment	^b^Adjusting for competing risk		
Variables	HR (95% CI)	*p* value	HR (95% CI)	*p* value
Age (years)	1.06 (1.05–1.07)	0.002	1.05 (1.04–1.06)	<0.0001^c^
Sex (female)	0.87(0.79–0.96)	0.007	0.89 (0.81–0.98)	0.02
*APOE* ε3ε3	Reference	‐	Reference	‐
*APOE* ε2ε2:ε2ε3	0.67(0.43–1.02)	0.06	0.99(0.85–1.15)	0.96
*APOE* ε3ε4:ε2ε4	1.35(1.20–1.51)	<0.0001^+^	1.35(1.20 −1.51)	<0.0001^+^
*APOE* ε4ε4	1.75(1.24–2.46)	0.001	1.74(1.22 −2.47)	0.001
Low PRS tertile	Reference	‐	Reference	‐
Middle PRS tertile	1.02(0.91–1.16)	0.64	1.03(0.91–1.16)	0.63
High PRS tertile	1.08(0.96–1.22)	0.18	1.10(0.95–1.21)	0.22

Abbreviations: CI, Confidence interval; HR, Hazard ratio; PRS, Polygenic risk score.

^a^ COX proportional hazard models.

^b^ Risk regression model by Fine and Gray Method.

^c^ Denotes *p* values = 1.0 × 10^−16^,+denotes *p* < 16.3 × 10^−12^.

Dementia risk was higher for participants in the high‐risk PRS tertile than the low (HR 1.36 [CI 1.0–1.7], *p *= 0.02) (Table 3a). CID in the high‐risk tertile was 9.6% (CI 7.8–11.8) compared with the low tertile 7.3% (CI 5.7–9.3) (Figure 1b; Table S5). At age 95 years, the effect of PRS was more prominent with CID increasing from 17.6% (13.4–23.0) in the low PRS tertile to 30.6% (21.9–41.9) in the high (Table S5).

In sub‐group analysis, among *APOE* ε3/ε4 heterozygotes, PRS modified dementia risk, with CID increasing from 10.8% (CI 7.2–16.3) in the low PRS tertile to 17.8% (CI 13.2–23.8) in the high (Table S6). Among *APOE* ε4/ε4 homozygotes with high PRS (the highest genetic risk category), CID was 32.2% (CI 11.3–71.6). In ε4/ε4 homozygotes with low PRS, CID was lower at 24.6% (CI 11.2–48.8). For ε3/ε3 homozygotes, CID in the low‐risk PRS tertile was 5.7% (CI 3.9–8.3) and in the high‐risk 7.6% (CI 5.5–10.5) (Table S6).

We compared CID between the highest genetic risk group at age 80 (ε4 carriers with high PRS) and the lowest genetic risk group at age 90 (ε2 carriers with low PRS). CID in the highest genetic risk group at age 80 was 6.1% (CI 4.1–9.0) and in the lowest genetic risk at age 90 was 8.8% (CI 4.5–16.7) (Table S6). This corresponded to an approximately 10‐year delay in age of onset between these two extreme groups. In sensitivity analysis, we examined interaction between *APOE*/PRS and found no significant association with incident dementia (*p *> 0.05).

**FIGURE 2:**
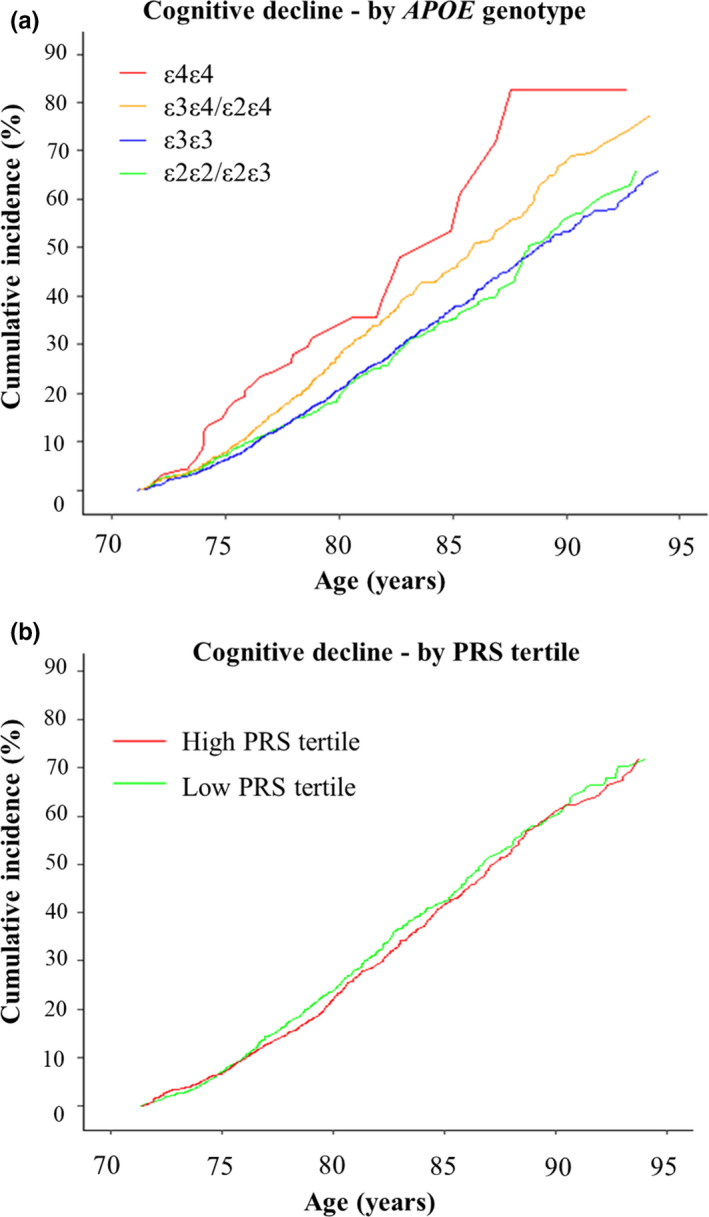
Cumulative incidence of cognitive decline stratified by *APOE* genotypes and tertiles of a polygenic risk score (PRS). Cumulative incidence curves for cognitive decline were calculated with mortality as a competing event, stratified by *APOE* genotypes (a) tertiles of a PRS (b) based on 23 common non‐*APOE* variants. High PRS is shown in red; low PRS in green (mid tertile not shown). Confidence intervals and participants at risk are shown in Table S6–7

A total of 1598 (12.6%) participants had cognitive decline (Table 1). The cumulative incidence of cognitive decline to age 85 years was estimated to be 37.2% (CI 36.4–41.0) in *APOE* ε3/ε3 homozygotes, 35.3% (CI 30.5–39.6) in ε2/ε2 homozygotes, 45.7% (CI 46.5–53.9) in ε3/ε4 heterozygotes and 52.9% (CI 46.1–76.2) in ε4/ε4 homozygotes (Figure 2a, Table S7). Compared with the ε3/ε3 reference group, cognitive decline risk was significantly higher in ε3/ε4 heterozygotes (HR = 1.35 [1.20–1.51], *p *< 0.001) and ε4 homozygotes (HR = 1.75 [CI 1.24–2.46]), *p *< 0.001) (Table 3b). PRS was not associated with cognitive decline. Risk of cognitive decline did not significantly increase between low and high PRS tertiles (HR = 1.08 [0.96–1.22], *p *= 0.18) (Figure 2b, Table S8‐S9). In sensitivity analysis, the interaction effect between *APOE*/PRS for cognitive decline was not significant (*p *> 0.05).

## 4 DISCUSSION

In this study, we examined the effect of *APOE* genotypes and PRS on incident dementia and cognitive decline among 12,978 initially healthy older participants. We found that *APOE* ε4 and high PRS were associated with increased relative risk of dementia, but overall, cumulative incidence of dementia was low across all genotype groups. PRS effect on dementia risk was modest and delayed compared with *APOE* ε4, mostly affecting risk after 85 years of age. *APOE* ε4 was associated with cognitive decline, but PRS was not, suggesting that *APOE* genotype has a stronger effect than PRS on both dementia and cognitive decline. We observe that the absence of co‐morbidities, atherothrombotic cardiovascular disease and cognitive impairment to age 70 years contributed to the attenuation of incident dementia across all genotypes.

The unique ascertainment of the ASPREE population is an important factor in the interpretation of our results. The eligibility criteria excluded individuals with dementia diagnoses and cognitive impairment at enrolment, and individuals with any history or diagnosis of atherothrombotic cardiovascular disease events, major physical disability or life‐threatening cancer (McNeil et al., 2018a). This produced a highly selected population of healthy older individuals, who at the time of study entry, benefited from the absence of several important dementia risk factors. This selective pressure resulted in deviation from the Hardy–Weinberg equilibrium, with fewer deleterious *APOE* ε4 alleles observed than expected. Selection against ε4 was driven by the age cut‐off (>70 years), as well as the strict inclusion/exclusion criteria of the trial.

We accordingly observed a low cumulative incidence of all‐cause dementia, estimated to be 7.4% to age 85 across all participants. This estimate was approximately half that reported in the community‐based Rotterdam study to the same age (15.6%) (Lee et al., 2018. While acknowledging potential issues with comparing dementia risk between different studies, including differences in population demography, recruitment criteria, diagnostic definitions and duration of follow‐up (Desikan et al., 2017; Qian et al., 2017; Lee et al., 2018), we consider comparisons between ASPREE and the Rotterdam study warranted. The studies had similar sample sizes, age ranges, sex percentages, diabetes, hypertension, BMI, blood lipids, genetic ancestry and adjudicated dementia cases. Further, both analyses used the same PRS calculations (Desikan et al., 2017; Lambert et al., 2013; Ruiz et al., 2014).

The lower risk of dementia in ASPREE is likely influenced by the selection of healthy participants, depletion of deleterious *APOE* ε4 alleles, and a relatively short follow‐up period where healthy selection effects have not yet dissipated. The estimated CID in ASPREE was 26.6% for ε4/ε4 homozygotes, compared with approximately 60% in the Rotterdam study, and 5.9% for ε3/ε4 heterozygotes, compared with approximately 25% in the Rotterdam study (Lee et al., 2018). These differences in CID are substantial, unlikely to be attributable to confounding factors alone between the studies.

Further, in a recent meta‐analysis of three population‐based cohorts of cognitively normal subjects aged 60–75 years (total *N* = 11,771), the risk of dementia in *APOE* ε4/ε4 homozygotes (*N* = 134) to age 70–75 years was 11.2% (Qian et al., 2017). In ASPREE, however, the risk of dementia to age 75 in ε4/ε4 (*N* = 200) was only 3.7%. Risk of dementia among ε4/ε4 homozygotes to age 85 years in the Framingham Heart Study (37.6%, *N* = 67) was also considerably higher than ASPREE (26.6% *N* = 200) (Qian et al., 2017). We acknowledge the variation in genetic risk of dementia between ethnic groups (Teruel et al., 2011), yet our study was not designed to assess ethnic differences. We assessed genetic effects in individuals of European ancestry only, and compared results with another similar sized cohort of European ancestry (the Rotterdam Study (Lee et al., 2018)). We did not include individuals with non‐European ancestry in the analysis, due to small sample size and the risk of population stratification bias influencing genetic risk estimates.

PRS is more challenging to interpret across studies, given the different PRSs used (Tan et al., 2017; Chouraki et al., 2016; Cruchaga et al., 2018; Desikan et al., 2017; Qian et al., 2017; Lee et al., 2018). However, we also observed an attenuated effect of PRS on dementia in ASPREE compared with other studies. We observed only a 2.6% difference in CID between low (7.3%) and high (9.6%) PRS tertiles. In the Rotterdam study, the observed difference was 9.0% between low (11.6%) and high (20.4%) tertiles to the same age.

In ASPREE, the effect of PRS was more pronounced in *APOE* ε4 carriers, compared with the reference ε3/ε3 group. However, the PRS effect was attenuated and delayed in age of onset compared with other studies (Peloso et al., 2020; Lee et al., 2018). The PRS effect on dementia risk in ASPREE mostly occurred after the age of 85 years (Figure 2a). We found no significant interaction effect between *APOE* and PRS in ASPREE, unlike the Rotterdam study (Lee et al., 2018). This again may reflect the attenuation of genetic effects on dementia risk in ASPREE. A recent analysis of the Framingham cohort also reported no significant interaction between *APOE* and PRS while evaluating dementia risk (Peloso et al., 2020). Further studies with large populations and longer follow‐up are required to understand interactions between *APOE* and PRS in modifying dementia risk. The majority of dementia events observed in our study were classified as either ‘probable AD’ or ‘possible AD’, with only 5 cases classified as ‘non‐AD related’. Therefore, we were unable to undertake sub‐group analysis, based on dementia sub‐classifications.

Considering the attenuated genetic risk of dementia observed in ASPREE, we query whether other factors further modified risk, beyond the low vascular risk, cognitive screening and absence of cardiovascular disease at baseline. Such factors could include a favourable lifestyle, characterised by healthy diet, regular exercise and high socialisation levels (Licher et al., 2019; Lourida et al., 2019). Alternatively, the attenuation could be related to the relatively short follow‐up period, during which healthy selection effects had not yet dissipated.

Protective genetic loci not included in the PRS may also have contributed to risk modification, including common variants yet to be identified by GWAS and/or rare high‐effect protective variants, including loss‐of‐function variants in biologically associated genes. There is growing evidence that protection from dementia risk can be conferred by both common and rare genetic variants, especially in the high‐risk *APOE* ε4/ε4 group (Belloy et al., 2020; Huq et al., 2019). Further studies are required to examine the effect of protective genetic variants for dementia in ASPREE.

*APOE* ε4 carrier status was significantly associated with cognitive decline in ASPREE, but PRS was not. This reflects the more modest effect of PRS on cognitive ageing, and/or a divergent genetic aetiology versus *APOE* genotype (Harris et al., 2014). The association between *APOE* ε4 and cognitive decline in non‐demented individuals has been reported by several studies using comparable cognitive testing (Albrecht et al., 2015; Jager et al., 2012; Reas et al., 2019; Verhaaren et al., 2013; Wisdom et al., 2011). However, few studies have reported a significant effect of PRS on cognitive decline alone (Chaudhury et al., 2019; Harris et al., 2014; Marden et al., 2016; Verhaaren et al., 2013). It appears that PRS derived from GWAS of diagnosed dementia/AD cases are not strong predictors of cognitive decline without dementia during ageing. However, our approach to quantifying cognitive decline may be insensitive or might reflect a different biological process. Alternatively, PRS derived from a GWAS of dementia/AD cases may reflect the functional impairment required for dementia diagnosis, rather than the cognitive aspects.

Strengths of the study include a well‐characterised longitudinal cohort with repeated cognitive assessments and dementia adjudication, genetic data for both *APOE* and PRS variants, longitudinal follow‐up to enable survival analysis for dementia and cognition, data available on covariates, adjudicated reports of causes of death to control for competing events and a large number of initially healthy elderly participants.

Limitations of the study include a shorter duration of follow‐up compared with other studies (Qian et al., 2017; Lee et al., 2018 (possibly insufficient to overcome a healthy volunteer effect) and limited event numbers in some rarer *APOE* genotype groups. We also used a broad definition of cognitive decline (CD), characterised by a 1.5 SD inter‐individual decline on any cognitive test/domain. This broad measure of CD did not report or quantitate the frequency of domain‐specific decline.

In conclusion, our study found that *APOE* genotypes and PRS effect the relative risk of dementia in a population of healthy older individuals followed prospectively. However, overall CID in the population was low across all genotype groups, reflecting the healthy nature of the population at enrolment. *APOE* ε4 had a stronger effect than PRS on dementia risk. *APOE* genotypes affected cognitive decline, whereas PRS did not. Prospective studies of initially healthy older participants with longer follow‐up periods are required to further understand the genetic risk of dementia and cognitive decline during ageing, and examine the predictive performance and clinical utility of PRS.

## CONFLICT OF INTEREST

Dr. Shah serves as a non‐compensated member of the Board of Directors of the Alzheimer's Association—Illinois Chapter. His institution, Rush University Medical Center, receives funds for his role as Site Principal Investigator or Site Sub‐Investigator for industry‐sponsored clinical trials and research studies involving Alzheimer's disease and dementia sponsored by Amylyx Pharmaceuticals, Inc., Eli Lilly & Co., Inc., Genentech, Inc., Merck & Co., Inc., Navidea Biopharmaceuticals, and Novartis Pharmaceuticals, Inc. Dr Sebra serves as Vice‐President of Technology Development at Sema4. Dr Schadt serves as Chief Executive Officer at Sema4. Dr Goate has consulted for Biogen AbbVie, GSK, Eisai and Denali Therapeutics. No other conflicts were reported.

## AUTHOR CONTRIBUTIONS

MR, JR, RW and PL: Genetic Study design, Data analysis and manuscript writing. SO, AH, JL, RW, RW, JR, RS, ES, AB, AM and JM: Clinical trial study design, sample collection, phenotyping and manuscript writing. JR, JT, AR, AG, RS, ES and AB: Manuscript writing, expert view and proof reading.

## Supporting information

Supplementary Material

## Data Availability

The data that support the findings of this study are available on request from the senior authors of the study (PL and MR).
