# Supplementary material for: Effect of APOE and a polygenic risk score on incident dementia and cognitive decline in a healthy older population
Source: Aging Cell. 2021 May 26;20(6):e13384. doi: 10.1111/acel.13384 (PMC8208779; doi:10.1111/acel.13384)
Supplement: Supplementary file 1 — Supplementary Material [file ACEL-20-e13384-s001.docx]

**Supplementary Material**

**Genotyping and Principal Component analysis**

We followed best practice genotyping and quality control (QC) protocols from Thermo Fisher, starting from raw intensity CEL files, we used a custom script designed for the the AXIOM PMDA array uing the human genome reference GRCh38 , to produce variant call files. We performed sample level QC using plink version 1.9, excluding samples for gender discordance (80 samples mismatched and excluded) using plink default F statistics threshold (≤ 0.2 female and ≥ 0.8 male), relatedness (124 indviduals excluded) using default PI-HAT threshold >0.025 to exclude one sample from each related pair. To estimate population structure in the ASPREE cohort we performed principal component analysis (PCA) using The 1000 Genomes Project as a reference population (24). Directly genotyped data from ASPREE and The 1000 Genomes Project 1K phase 3 (liftover to GRCh38) were merged and LD pruned (r^2^ < 0.1) using plink version 1.9 (27), followed by R package SNPrelate (25). We calculated the Z score for first 2 principal component eigenvectors and excluded samples with ± 2SD (standard deviation) of Z score compared to their respective five reference superpopulation groups from the 1000 Genomes Project that included: Europenas, South Asians, East Asians, African American (African super population) and Hispanics (Ad Mixed American) (Figure S2). ASPREE samples from participants of non-European ancestry (N= 759) were not included in the final anlaysis (238 African American, 58 East Asian, 388 Hispanics and 75 South Asians), in order to minimize the risk of populaiton stratification bias in genetic risk estimates, and due to the relatively small sample size of particpants in these groups. The final dataset of 12,978 Non-Finish Europeans partiicpants was used for the subsequent analysis

Figure S1: Study flow chart indicating the selection of the final dataset.


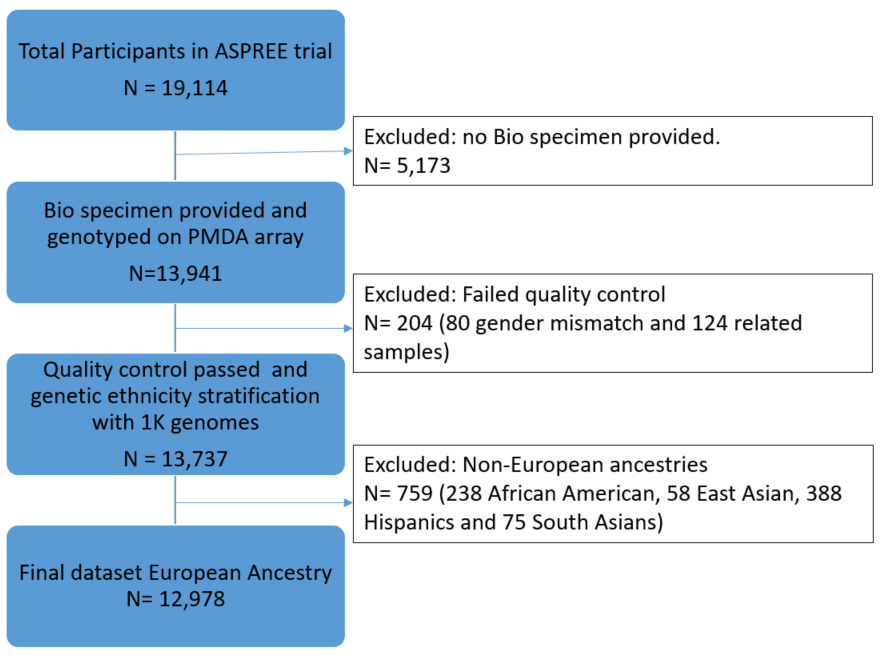


**Figure S2: Principal component analysis (PCA) of the ASPREE cohort compared with the 1000 Genomes Project.** **A)** PCA plot of all ASPREE participants projected onto 1000 Genome populations. **B).** PCA plot of ASPREE Europeans samples projected onto 1000 Genome Europeans samples that were included in this study. In Figure legend 1K genome populations are (Europeans, South Asians, East Asians, African American and Hispanics. ASPREE_AA is African American samples.


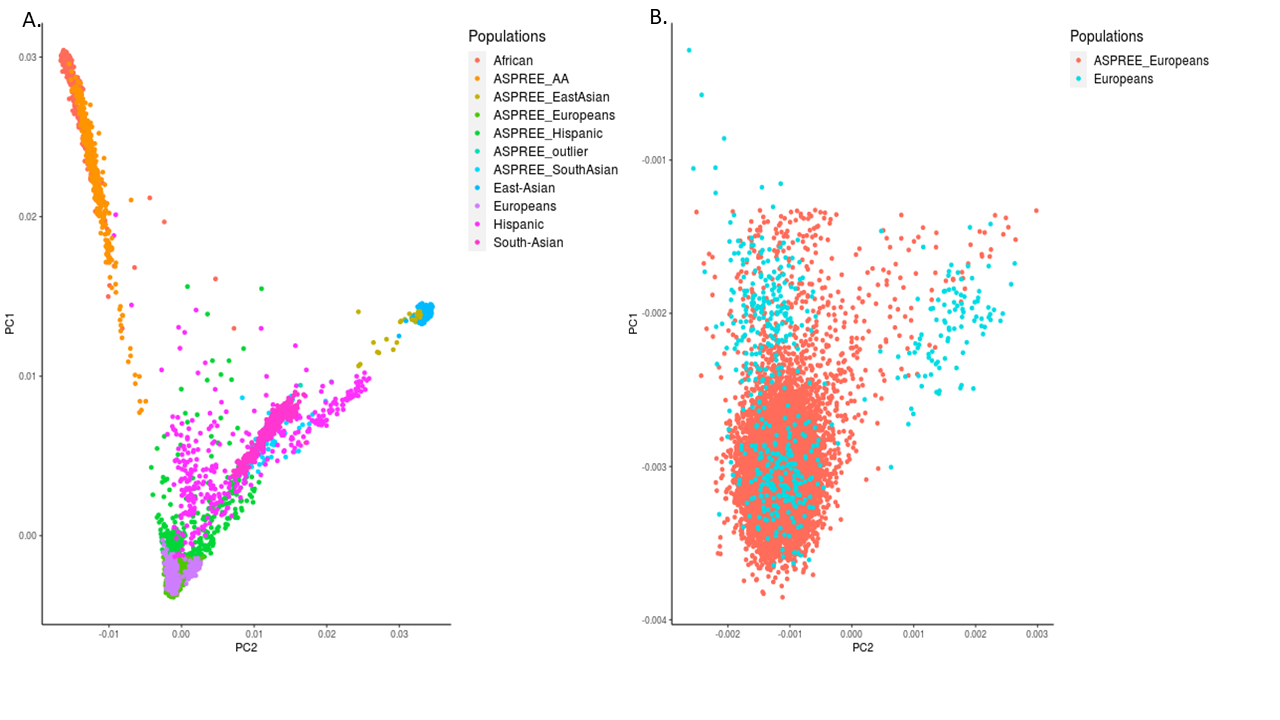


**Table S1: SNPs used in polygenic risk score (PRS) of dementia**

| Gene (NCBI) | SNP | Effect allele | | Effect Size (Beta)* | Reference No |
| --- | --- | --- | --- | --- | --- |
| ABCA7 | rs4147929 | G | -0·135 | | 4 |
| BIN1 | rs6733839 | T | 0·188 | | 4 |
| CASS4 | rs7274581 | C | -0·139 | | 4 |
| CD2AP | rs10948363 | G | 0·098 | | 4 |
| CELF1 | rs10838725 | C | 0·075 | | 4 |
| CLU | rs9331896 | T | 0·146 | | 4 |
| CR1 | rs6656401 | G | -0·157 | | 4 |
| ECHDC3 | rs7920721 | G | -0·067 | | 20 |
| EPHA1 | rs11771145 | A | -0·102 | | 4 |
| FERMT2 | rs17125944 | C | 0·122 | | 4 |
| HLA-DRB1/5 | rs111418223 merged into rs9271192 | A | -0·108 | | 4 |
| HS3ST1 | rs13113697 | G | -0·067 | | 20 |
| INPP5D | rs35349669 | T | 0·066 | | 4 |
| KANSL1 | rs118172952 merged into rs2732703 | G | -0·151 | | 4 |
| MEF2C | rs190982 | A | 0·08 | | 4 |
| MS4A6A | rs983392 | G | -0·108 | | 4 |
| NME8 | rs2718058 | G | -0·07 | | 4 |
| PICALM | rs10792832 | G | 0·13 | | 4 |
| PTK2B | rs28834970 | C | 0·096 | | 4 |
| SLC24A4-RIN3 | rs10498633 | T | -0·104 | | 4 |
| SORL1 | rs11218343 | C | -0·27 | | 4 |
| TREM2 | rs75932628 | T | 0·889 | | 21 |
| ZCWPW1 | rs1476679 | T | 0·078 | | 4 |
| *Shows the weighted effect size used by *Lee et al 2018* (9) to calculate the PRS. | | | | | |

**Table S2:** STROBE Statement—Checklist of items that should be included in reports of ***cohort studies***

|  | Item No | Recommendation | Page No |
| --- | --- | --- | --- |
| **Title and abstract** | 1 | (*a*) Indicate the study’s design with a commonly used term in the title or the abstract |  |
|  |  | (*b*) Provide in the abstract an informative and balanced summary of what was done and what was found | 2, |
| Introduction | | | |
| Background/rationale | 2 | Explain the scientific background and rationale for the investigation being reported | 4 |
| Objectives | 3 | State specific objectives, including any prespecified hypotheses | 5 |
| Methods | | | |
| Study design | 4 | Present key elements of study design early in the paper | 6,7 |
| Setting | 5 | Describe the setting, locations, and relevant dates, including periods of recruitment, exposure, follow-up, and data collection | 6-8 |
| Participants | 6 | (*a*) Give the eligibility criteria, and the sources and methods of selection of participants. Describe methods of follow-up | 6-9 |
|  |  | (*b*) For matched studies, give matching criteria and number of exposed and unexposed |  |
| Variables | 7 | Clearly define all outcomes, exposures, predictors, potential confounders, and effect modifiers. Give diagnostic criteria, if applicable | 8-9 |
| Data sources/ measurement | 8* | For each variable of interest, give sources of data and details of methods of assessment (measurement). Describe comparability of assessment methods if there is more than one group | 8-9 |
| Bias | 9 | Describe any efforts to address potential sources of bias | 6-9 and Supplement material |
| Study size | 10 | Explain how the study size was arrived at | 7 and Figure S1 Supplement material |
| Quantitative variables | 11 | Explain how quantitative variables were handled in the analyses. If applicable, describe which groupings were chosen and why | 8-9 |
| Statistical methods | 12 | (*a*) Describe all statistical methods, including those used to control for confounding | 6-9 |
|  |  | (*b*) Describe any methods used to examine subgroups and interactions |  |
|  |  | (*c*) Explain how missing data were addressed |  |
|  |  | (*d*) If applicable, explain how loss to follow-up was addressed |  |
|  |  | (*e*) Describe any sensitivity analyses |  |
| Results | | |  |
| Participants | 13* | (a) Report numbers of individuals at each stage of study—eg numbers potentially eligible, examined for eligibility, confirmed eligible, included in the study, completing follow-up, and analysed | 10-12 |
|  |  | (b) Give reasons for non-participation at each stage |  |
|  |  | (c) Consider use of a flow diagram |  |
| Descriptive data | 14* | (a) Give characteristics of study participants (eg demographic, clinical, social) and information on exposures and potential confounders | 23-27 |
|  |  | (b) Indicate number of participants with missing data for each variable of interest |  |
|  |  | (c) Summarise follow-up time (eg, average and total amount) |  |
| Outcome data | 15* | Report numbers of outcome events or summary measures over time | 10, 11 and 23-27 |

| Main results | 16 | (*a*) Give unadjusted estimates and, if applicable, confounder-adjusted estimates and their precision (eg, 95% confidence interval). Make clear which confounders were adjusted for and why they were included | 23-27  and Supplementary Tables S2-S5 |
| --- | --- | --- | --- |
|  |  | (*b*) Report category boundaries when continuous variables were categorized |  |
|  |  | (*c*) If relevant, consider translating estimates of relative risk into absolute risk for a meaningful time period |  |
| Other analyses | 17 | Report other analyses done—eg analyses of subgroups and interactions, and sensitivity analyses | Supplementary Tables S2-S9 |
| Discussion | | | |
| Key results | 18 | Summarise key results with reference to study objectives | 13 |
| Limitations | 19 | Discuss limitations of the study, taking into account sources of potential bias or imprecision. Discuss both direction and magnitude of any potential bias | 16 |
| Interpretation | 20 | Give a cautious overall interpretation of results considering objectives, limitations, multiplicity of analyses, results from similar studies, and other relevant evidence | 13-15 |
| Generalisability | 21 | Discuss the generalisability (external validity) of the study results | 16 |
| Other information | | | |
| Funding | 22 | Give the source of funding and the role of the funders for the present study and, if applicable, for the original study on which the present article is based | 17 |

*Give information separately for exposed and unexposed groups.

**Note:** An Explanation and Elaboration article discusses each checklist item and gives methodological background and published examples of transparent reporting. The STROBE checklist is best used in conjunction with this article (freely available on the Web sites of PLoS Medicine at http://www.plosmedicine.org/, Annals of Internal Medicine at http://www.annals.org/, and Epidemiology at http://www.epidem.com/). Information on the STROBE Initiative is available at http://www.strobe-statement.org.

**Table S3.** **Hardy-Weinberg equilibrium (HWE) testing for *APOE* genotype frequencies in the ASPREE population.** The chi square test was performed to calculate deviation from Hardy-Weinberg equilibrium [Preacher, K. J. (2001, April). Calculation for the chi-square test: An interactive calculation tool for chi-square tests of goodness of fit and independence [Computer software]. Available from http://quantpsy.org.]

| **Alleles: P** = 0.77 (*APOE* ε3), **Q** = 0.13 (*APOE* ε4), **R** = 0.09 (*APOE* ε2) | | | | |
| --- | --- | --- | --- | --- |
|  | **Observed** | **Expected** | **Chi Square** | **P value** |
| ε3ε3 | 7800 | 7743 | 38.0 | P < 0.001 |
| ε3ε4 | 2665 | 2723 |  |  |
| ε2ε2 | 68 | 109 |  |  |
| ε2ε3 | 1784 | 1839 |  |  |
| ε2ε4 | 461 | 323 |  |  |
| ε4ε4 | 200 | 239 |  |  |

**Figure S3: Polygenic risk score (PRS) distribution in the ASPREE cohort.** The red dotted line shows boundaries of PRS tertiles; lower risk tertile -0.56 (range: -1.43 to -0.34), middle risk tertile -0.20 (range -0.34 to -0.06) and high risk tertile 0.16 (range -0.06 to 1.86).


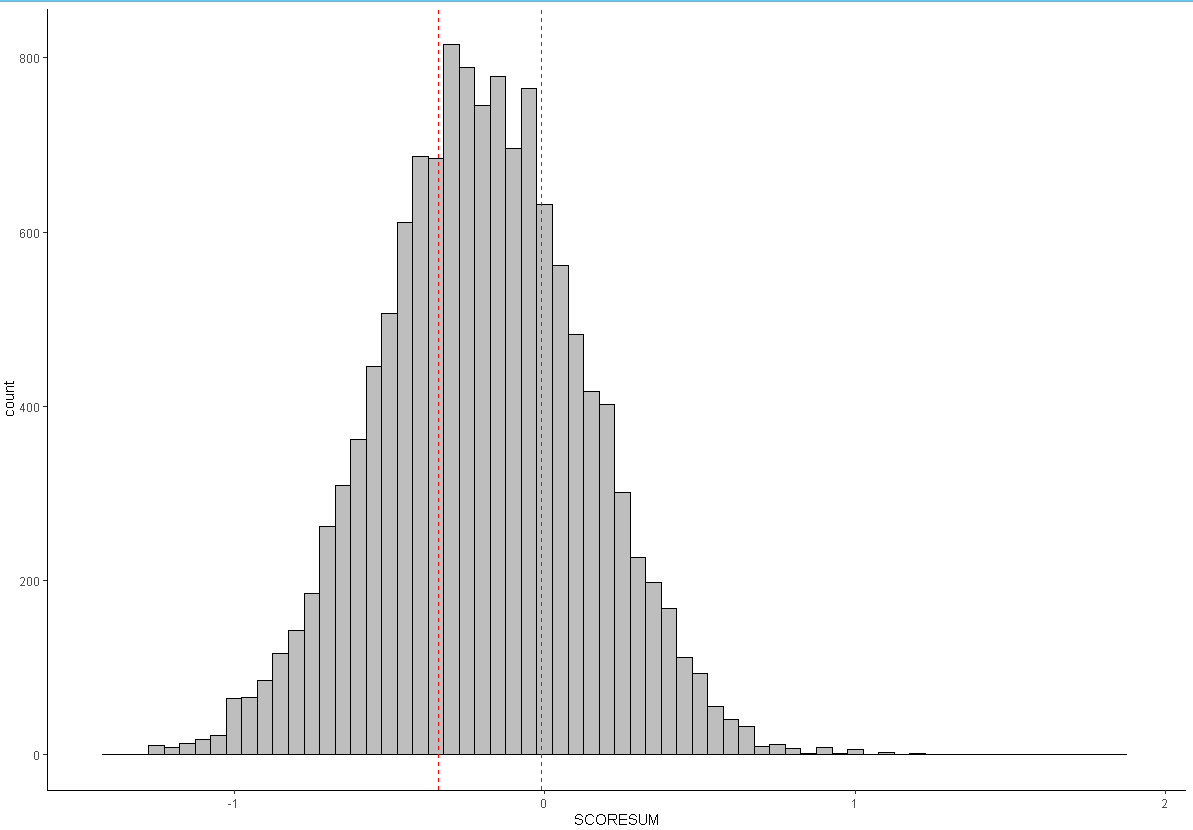


**Table S4: Cumulative incidence of dementia stratified by *APOE* genotypes, accounting for death as a competing risk**

| *APOE* genotypes | Age, years* | Participants at risk  N | Risk, [95% Confidence Interval] |
| --- | --- | --- | --- |
| ε2/ε2:ε2/ε3 | 75 | 835 | 0.2% [0.0-1.1] |
| ε3/ε3 | 75 | 3672 | 0.4% [0.2-0.7] |
| ε2/ε4:ε3/ε4 | 75 | 1555 | 1.0% [0.6-1.6] |
| ε4/ε4 | 75 | 103 | 3.7% [1.4-9.5] |
|  |  |  |  |
| ε2/ε2:ε2/ε3 | 80 | 462 | 1.2% [0.6-2.4] |
| ε3/ε3 | 80 | 1838 | 2.0% [1.5-2.6] |
| ε2/ε4:ε3/ε4 | 80 | 719 | 4.9% [3.8-6.3] |
| ε4/ε4 | 80 | 40 | 19.3% [11.9–30.5] |
|  |  |  |  |
| ε2/ε2:ε2/ε3 | 85 | 217 | 4.0% [2.4-6.5] |
| ε3/ε3 | 85 | 762 | 5.9% [4.8-7.2] |
| ε2/ε4:ε3/ε4 | 85 | 258 | 12.6% [10.2-15.5] |
| ε4/ε4 | 85 | 17 | 26.6% [16.2–42.0] |
|  |  |  |  |
| ε2/ε2:ε2/ε3 | 90 | 38 | 8.7% [5.4-14.1] |
| ε3/ε3 | 90 | 193 | 5.9% [4.8-7.2] |
| ε2/ε4:ε3/ε4 | 90 | 58 | 12.6% [10.2-15.5] |
| ε4/ε4 | 90 | 3 | 26.6% [16.2–42.0] |
|  |  |  |  |
| ε2/ε2:ε2/ε3 | 95 | 7 | 15.4% [6.5-34.0] |
| ε3/ε3 | 95 | 17 | 21.2% [15.0-2.5] |
| ε2/ε4:ε3/ε4 | 95 | 5 | 42.0% [28.7-58.4] |
| ε4/ε4 | 95 | 1 | 48.5% [20.5–85.3] |
| *Five-year longitudinal age from baseline to dementia or censored | | | |
|  |  |  |  |

**Table S5: Cumulative incidence of dementia stratified by tertiles of a polygenic risk score (PRS), accounting for death as a competing risk**

| PRS Tertiles | Age, years | Participants at risk  N | Risk, [95% Confidence Interval] |
| --- | --- | --- | --- |
| Low | 75 | 2038 | 0.6% [0.3–1.1] |
| Middle | 75 | 2024 | 0.7% [0.4-1.2] |
| high | 75 | 2096 | 0.4% [0.2-0.8] |
|  |  |  |  |
| Low | 80 | 1020 | 2.7% [2.0–3.6] |
| Middle | 80 | 1030 | 3.0% [2.2-4.0] |
| high | 80 | 1021 | 2.9% [2.2-3.9] |
|  |  |  |  |
| Low | 85 | 410 | 7.3% [5.7–9.3] |
| Middle | 85 | 429 | 5.8% [4.4-7.5] |
| high | 85 | 411 | 9.6% [7.8-11.8] |
|  |  |  |  |
| Low | 90 | 116 | 14.7% [11.5–18.6] |
| Middle | 90 | 88 | 15.3% [11.6-19.9] |
| high | 90 | 9 | 17.6% [14.2-21.8] |
|  |  |  |  |
| Low | 95 | 12 | 17.6% [13.4–23.0] |
| Middle | 95 | 8 | 27.8% [16.9-43.4] |
| high | 95 | 9 | 30.6% [21.9-41.9] |
| *Five-year longitudinal age from baseline to dementia or censored | | | |
|  |  |  |  |

**Table S6: Cumulative incidence of dementia stratified by *APOE* genotypes and tertiles of a PRS**

|  |  | **APOE:e3e3** |  | **APOE:e3e4ande2e4** | |  | **APOE:e2e2&e2e3** | |  | **APOE:e4e4** |  |
| --- | --- | --- | --- | --- | --- | --- | --- | --- | --- | --- | --- |
| **PRS Group** | **Age** | **Risk, 95% CI** | **N** | **Risk, 95% CI** | **N** | | **Risk, 95% CI** | **N** | | **Risk, 95% CI** | **N** |
| Low tertile | 75 | 0.6%, 0.2 – 1.2 | 1200 | 0.6%, 0.2 – 1.9 | 534 | | 0.9%, 0.2 – 3.5 | 270 | | 5.4%, 1.4 – 20.0 | 36 |
| Middle tertile | 75 | 0.3%, 0.1 – 1.0 | 1197 | 1.5%, 0.7 – 3.1 | 513 | | 0.0%, 0.0 -0.0 | 282 | | 6.1%, 1.6 – 22.5 | 36 |
| High tertile | 75 | 0.2%, 0.1 – 0.9 | 1277 | 1.0%, 0.4 – 2.4 | 505 | | 0.0%, 0.0 -0.0 | 283 | | 0.0%, 0.0 – 0.0 | 33 |
| Low tertile | 80 | 2.0%, 1.3 – 3.1 | 606 | 4.1%, 2.5 – 6.8 | 237 | | 2.1%, 0.8 – 5.5 | 147 | | 18.2%, 7.7 – 39.7 | 15 |
| Middle tertile | 80 | 2.3%, 1.5 – 3.6 | 608 | 4.6%, 3.0 – 7.1 | 251 | | 0.4%,0.1 -2.8 | 144 | | 23.6%, 11.9 – 43.4 | 22 |
| High tertile | 80 | 1.8%, 1.1 – 2.9 | 595 | 6.1%, 4.1 – 9.0 | 237 | | 1.2%, 0.4 – 3.6 | 166 | | 16.6%, 5.3 – 45.3 | 12 |
| Low tertile | 85 | 5.7%, 3.9 – 8.3 | 260 | 10.8%, 7.2 – 16.3 | 77 | | 7.1%, 3.6 – 13.7 | 66 | | 24.6%, 11.2 – 48.8 | 9 |
| Middle tertile | 85 | 4.8%, 3.2 – 7.0 | 266 | 9.1%, 6.0 – 13.8 | 95 | | 3.4%, 1.2 – 9.3 | 68 | |  |  |
| High tertile | 85 | 7.6%, 5.5 – 10.5 | 240 | 17.8%, 13.2 – 23.8 | 90 | | 2.9%, 1.1 – 7.4 | 77 | | 32.2%, 11.3 -71.6 | 5 |
| Low tertile | 90 | 11.6%, 8.1 – 16.6 | 80 | 24.4%, 16.9 – 34.5 | 28 | | 8.8%, 4.5 – 16.7 | 17 | |  |  |
| Middle tertile | 90 | 14.4%, 9.6 – 21.5 | 53 | 21.6%, 14.8 – 30.9 | 22 | | 7.9%, 3.2 – 18.8 | 6 | |  |  |
| High tertile | 90 | 15.2%, 11.1 – 20.6 | 67 | 32.3%, 22.8 – 44.3 | 15 | | 9.6%, 3.8 – 23.1 | 16 | |  |  |
| Low tertile | 95 | 14.0%, 9.6 – 20.2 | 8 | 31.9%, 19.0 – 50.4 | 6 | | 8.8%, 4.5 – 16.7 | 6 | |  |  |
| Middle tertile | 95 | 28.5%, 14.5 – 51.2 | 6 | 32.3%, 21.1 – 47.3 | 8 | | 7.9%, 3.2 – 18.8 | 2 | |  |  |
| High tertile | 95 | 21.3%, 15.4 – 29.3 | 7 | 76.8%, 61.4 – 89.4 | 1 | | 19.5%, 7.0 – 47.7 | 4 | |  |  |
| APOE: Apolipoprotein E, Dementia risk is determine using 5 years of age intervals accounted for death as competing risk, The cumulative incidence was calculated up to 95 years of age, beyond age 95 the data was very limited with no participant alive or demented also data was also sparse, similar case for APOE e4e4 beyond age 85 . N is number of participants at risk is for APOE categories for each PRS tertile. Empty cells show data was sparse or that the participants at those ages and APOE/PRS categories were not demented or alive. | | | | | | | | | | | |

**Table S7: Cumulative incidence of cognitive decline stratified by *APOE* genotypes, accounting for death as a competing risk**

| *APOE* genotypes | Age, years* | Participants at risk  N | Risk, [95% Confidence Interval] |
| --- | --- | --- | --- |
| ε2/ε2:ε2/ε3 | 75 | 668 | 7.3% [5.4-9.8] |
| ε3/ε3 | 75 | 2966 | 6.2% [5.4-7.2] |
| ε2/ε4:ε3/ε4 | 75 | 1233 | 7.8% [6.3-9.5] |
| ε4/ε4 | 75 | 76 | 15.9 %[9.7-25.4] |
|  |  |  |  |
| ε2/ε2:ε2/ε3 | 80 | 352 | 19.0% [16.1-22.4] |
| ε3/ε3 | 80 | 1346 | 20.8% [19.2-22.4] |
| ε2/ε4:ε3/ε4 | 80 | 504 | 27.5% [24.9-30.4] |
| ε4/ε4 | 80 | 34 | 33.1% [23.6–45.1] |
|  |  |  |  |
| ε2/ε2:ε2/ε3 | 85 | 155 | 35.3% [30.5-39.6] |
| ε3/ε3 | 85 | 553 | 37.2% [36.4-41.0] |
| ε2/ε4:ε3/ε4 | 85 | 174 | 45.7% [46.5-53.9] |
| ε4/ε4 | 85 | 9 | 52.9% [46.1–76.2] |
|  |  |  |  |
| ε2/ε2:ε2/ε3 | 90 | 26 | 54.9% [48.4-61.7] |
| ε3/ε3 | 90 | 134 | 52.9% [49.7-56.1] |
| ε2/ε4:ε3/ε4 | 90 | 33 | 67.3% [62.0-72.4] |
| ε4/ε4 | 90 | 2 | 81.8% [59.6-95.9] |
|  |  |  |  |
| ε2/ε2:ε2/ε3 | 95 | 6 | 63.7% [55.2-72.1] |
| ε3/ε3 | 95 | 12 | 64.4% [59.8 -68.9] |
| ε2/ε4:ε3/ε4 | 95 | 4 | 75.1% [68.0-81.7] |
| ε4/ε4 | 95 | 1 | 81.8% [59.6-95.9] |
| *Five-year longitudinal age from baseline to cognitive decline or censored | | | |
|  |  |  |  |

**Table S8: Cumulative incidence of cognitive decline stratified by tertiles of a polygenic risk score (PRS), accounting for death as a competing risk**

| PRS Tertiles | Age, years* | Participants at risk  N | Risk, [95% Confidence Interval] |
| --- | --- | --- | --- |
| Low | 75 | 1662 | 6.7% [5.5-8.1] |
| Middle | 75 | 1608 | 6.8% [5.6-8.2] |
| high | 75 | 1677 | 7.3% [6.1-8.7] |
|  |  |  |  |
| Low | 80 | 748 | 21.5% [19.4-23.8] |
| Middle | 80 | 752 | 22.7% [20.5-25.0] |
| high | 80 | 730 | 22.9% [20.8-25.2] |
|  |  |  |  |
| Low | 85 | 283 | 38.9% [35.8-42.2] |
| Middle | 85 | 315 | 38.7% [35.7-41.9] |
| high | 85 | 290 | 39.8% [36.7-43.0] |
|  |  |  |  |
| Low | 90 | 75 | 57.0% [52.7-61.4] |
| Middle | 90 | 60 | 58.2% [53.7-62.8] |
| high | 90 | 65 | 55.5% [51.4-59.8] |
|  |  |  |  |
| Low | 95 | 4 | 66.3% [60.5-72.0] |
| Middle | 95 | 2 | 74.5% [63.9-83.9] |
| high | 95 | 6 | 66.8% [61.0-72.4] |
| *Five-year longitudinal age from baseline to cognitive decline or censored | | | |

**Table S9: Cumulative incidence of cognitive decline stratified by *APOE* genotypes and tertiles of a PRS**

|  |  | **APOE:e3e3** |  | **APOE:e3e4ande2e4** | |  | **APOE:e2e2&e2e3** |  | **APOE:e4e4** |  |
| --- | --- | --- | --- | --- | --- | --- | --- | --- | --- | --- |
| **PRS Group** | **Age** | **Risk, 95% CI** | **N** | **Risk, 95% CI** | **N** | | **Risk, 95% CI** | **N** | **Risk, 95% CI** | **N** |
| Low tertile | 75 | 5.9%, 4.5 – 7.7 | 987 | 6.3%, 4.3 – 9.3 | 422 | | 8.9%, 5.4 – 14.5 | 223 | 27.5%, 14.6 – 48.1 | 27 |
| Middle tertile | 75 | 6.2%, 4.8 – 8.1 | 961 | 7.8%, 5.4 – 11.1 | 401 | | 7.5%, 4.6 – 12.0 | 228 | 15.8%, 6.3 – 36.9 | 24 |
| High tertile | 75 | 6.6%, 5.2 – 8.4 | 1029 | 9.5%, 6.9 – 13.1 | 401 | | 6.2%, 3.6 – 10.6 | 223 | 8.6%, 2.8 – 24.3 | 28 |
| Low tertile | 80 | 19.6%, 17.0 – 22.5 | 462 | 25.9%, 21.5 – 31.0 | 169 | | 19,4%, 14.3 – 26.0 | 114 | 46.0%, 29.6 – 66.1 | 12 |
| Middle tertile | 80 | 21.1%, 18.5 – 24.1 | 460 | 28.1%, 23.5 – 33.3 | 169 | | 20.4%,15.5 – 26.7 | 110 | 31.9%, 15.9 – 57.2 | 10 |
| High tertile | 80 | 21.5%, 18.9 – 24.5 | 431 | 29.4%, 24.8 – 34.5 | 163 | | 18.1%, 13.5 – 24.0 | 127 | 49.7%, 19.7 – 88.5 | 3 |
| Low tertile | 85 | 37.8%, 33.8 – 42.1 | 187 | 43.7%, 37.0 – 50.9 | 47 | | 35.7%, 28.2 – 44.5 | 44 | 52.0%, 34.1 – 72.6 | 6 |
| Middle tertile | 85 | 36.6%, 32.8 – 40.6 | 194 | 47.0%, 40.8 – 53.6 | 66 | | 33.2%, 26.1 – 41.6 | 54 | 72.7%, 34.0 – 98.3 | 2 |
| High tertile | 85 | 37.6%, 33.6 – 41.8 | 171 | 46.6%, 40.3 – 53.3 | 64 | | 38.1%, 31.0 – 46.4 | 54 | 73.2%, 35.9 – 98.0 | 2 |
| Low tertile | 90 | 55.8%, 50.4 – 61.4 | 53 | 63.4%, 54.1 – 72.7 | 11 | | 55.0%, 44.0 – 67.2 | 13 | 52.0%, 34.2 – 72.6 | 6 |
| Middle tertile | 90 | 52.7%, 47.1 – 58.6 | 39 | 71.5%, 62.8 – 79.8 | 13 | | 53.6%, 42.8 – 65.1 | 5 |  |  |
| High tertile | 90 | 50.9%, 45.7 – 56.4 | 49 | 68.8%, 59.5 – 77.7 | 10 | | 55.0%,44.3 – 65.8 | 9 | 96.7%, 85.7 – 99.8 | 1 |
| Low tertile | 95 | 66.0%, 58.4 – 73.5 | 3 | 65.6%, 56.1 – 75.0 | 2 | | 66.1%, 52.7 – 79.1 | 4 |  |  |
| Middle tertile | 95 | 80.0%, 73.4 – 85.9 | 1 | 79.6%, 68.2 – 89.0 | 2 | | 53.6%, 42.8 – 65.1 | 4 |  |  |
| High tertile | 95 | 63.1%, 55.4 – 70.8 | 5 | 77.7%, 65.8 – 87.8 | 2 | | 63.4%, 50.4 – 76.2 | 4 |  |  |
| APOE: Apolipoprotein E, Cognitive decline incidence is measured using 5 years of age intervals accounted for death as a competing risk. The cumulative incidence was calculated up to 95 years of age, beyond age 95 the data was limited N is the number of participants at risk APOE categories for each PRS tertile, Empty cells show data was sparse or that the participants at those ages and APOE/PRS categories were not cognitive declined or alive. | | | | | | | | | | |
